# Longitudinal Residential Eating Disorder Treatment Outcomes for Binge‐Eating Disorder When Using the Unified Treatment Model

**DOI:** 10.1002/eat.70143

**Published:** 2026-07-06

**Authors:** Kiki M. Kline, Briana Delibashi, Gayle Brooks, Shelby N. Ortiz

**Affiliations:** ^1^ The Renfrew Center Coconut Creek Florida USA; ^2^ The Renfrew Center Philadelphia Pennsylvania USA; ^3^ Department of Psychiatry University of Michigan Ann Arbor Michigan USA

**Keywords:** anxiety, binge eating, binge eating disorder, depression, eating disorder psychopathology, eating disorders, longitudinal, outcomes research, residential treatment, unified treatment model

## Abstract

**Objective:**

Despite its prevalence, research on binge‐eating disorder (BED) outcomes for residential treatment remains limited. To address this research scarcity, we evaluated changes in ED behaviors and comorbid symptoms, including depression and anxiety, for patients with BED that received residential eating disorder (ED) care from a transdiagnostic ED treatment organization in the United States at the time of admission, discharge, and 6 months following discharge from treatment.

**Method:**

Participants were 168 female‐identifying patients diagnosed with BED at the time of admission to ED residential treatment. Patients completed measures of ED, anxiety, and depressive symptoms at admission, discharge, and 6 months following residential treatment. They also reported the number of binge‐eating episodes over the past 28 days at each time point.

**Results:**

Using linear mixed model analyses to compare changes in symptoms across the three time points, results revealed a statistically significant decrease in ED, anxiety, and depressive symptoms and binge‐eating behaviors from admission to discharge (Cohen's *dz* = 1.36, 1.04, 1.06, and 0.37, respectively) and from admission to 6‐month follow‐up (Cohen's *dz* = 0.64, 2.80, 0.86, and 0.16, respectively). There was a statistically significant rebound in symptoms from discharge to 6‐month follow‐up for ED (Cohen's *dz* = −0.59) and anxiety symptoms (Cohen's *dz* = −1.37) and binge‐eating behaviors (Cohen's *dz* = −0.69), but not for depressive symptoms (Cohen's *dz* = −0.13).

**Discussion:**

While partial symptom resurgence occurred for most symptoms at the 6‐month follow‐up time point, scores remained statistically lower than admission scores. These findings highlight that residential treatment may be effective for treating patients with BED, but direct comparisons with conditions are required.

## Summary

- Despite its prevalence, research on binge‐eating disorder outcomes for residential eating disorder treatment programs remains limited.
- This study demonstrated that using the Unified Treatment Model may be effective for treating patients with binge‐eating disorder.
- Findings revealed significant long‐term decreases in binge eating episodes and eating disorder, anxiety, and depressive symptoms from admission to discharge and at 6‐month follow‐up.
- While partial symptom resurgence occurred for most symptoms at the 6‐month follow‐up time point, scores remained statistically lower than admission scores.

## 1 Introduction

Binge‐eating disorder (BED), the most prevalent eating disorder (ED) worldwide (Qian et al. 2022), is characterized by a perceived loss of control during episodes of consuming an objectively large amount of food within a discrete period of time (i.e., binge eating [BE]; American Psychiatric Association [APA] 2022). However, rates of receiving ED care, including residential care, are extremely low compared to other EDs (Ali et al. 2020). Research on residential ED treatment indicates that only a small proportion of individuals receiving care are diagnosed with BED, compared to other EDs such as anorexia nervosa (AN), bulimia nervosa (BN), other specified feeding and eating disorders (OSFED) and eating disorders not otherwise specified (EDNOS; Fewell et al. 2017; Rienecke et al. 2024; Thompson‐Brenner et al. 2018; Vanderlinden et al. 2021). In many residential settings, individuals with BED receive interventions primarily aimed at weight loss (e.g., Giordano et al. 2017; Calugi et al. 2016; Kolotkin et al. 2004; Bonifacio et al. 2017), rather than mental health‐focused or ED‐specific treatment (APA 2023). The purpose of this study was to examine the treatment outcomes of patients diagnosed with BED who attended a residential ED treatment program in the United States.

Outpatient treatment is often the first‐line level of care for individuals with BED (Hilbert et al. 2012). However, when patients do not achieve adequate symptom improvement in outpatient settings, residential treatment specifically addressing ED symptoms may be warranted (APA 2023). Historically, the need for residential care for individuals with BED has been underrecognized. Because BED is not typically associated with the acute medical risks seen in disorders such as AN or BN, the considerable emotional, psychological, and functional impairments characteristic of BED are often underestimated as needing 24‐h monitoring and support (Lewinsohn et al. 2000; Kessler et al. 2013, 2014). Other barriers to this level of care include insufficient awareness of specialized treatment options (Supina et al. 2016; de Moraes et al. 2023) and insurance‐related barriers to access (Hart et al. 2011).

Residential treatment may be particularly beneficial for interrupting compulsive BE cycles in a structured environment, addressing functional impairments in daily living, and managing severe psychiatric comorbidity. Nationally representative data from adults in the United States demonstrate the high co‐occurrence of BED with depressive disorders (70%), anxiety disorders (59%), and post‐traumatic stress disorder (32%; Udo and Grilo 2019). Additionally, safety concerns are prevalent, with approximately 31% of individuals with BED reporting suicidal ideation or attempts (Bulik et al. 2021). Residential treatment has been shown to be effective in managing these symptoms for patients with EDs. These programs often integrate transdiagnostic and trauma‐specific therapeutic modalities such as cognitive behavioral therapy, acceptance and commitment therapy, and cognitive processing therapy as part of the treatment protocol, which is effective in addressing an array of emotional and distressing mental health symptoms (De Boer et al. 2023; Rienecke et al. 2024; Peckmezian and Paxton 2020; Brewerton et al. 2023).

### 1.1 Research on BED in Residential Care

There is a paucity of research specifically examining outcomes for individuals with BED in residential ED treatment settings. A systematic review of residential ED treatment outcomes found that only 14% of eligible studies included participants with BED, compared with 100% for AN, 90% for BN, and 71% for EDNOS/OSFED (Peckmezian and Paxton 2020). Further, much of the existing literature combined BED with other ED diagnoses, often due to the small number of participants with BED (e.g., Fewell et al. 2017; Rienecke et al. 2024; Thompson‐Brenner et al. 2018). Finally, to date, all of the existing literature has only used samples located in the United States.

Despite these limitations, the emerging evidence suggests that residential treatment outcomes for BED are promising (Peckmezian and Paxton 2020). Individuals with BED may respond well and benefit quickly from residential care that does not focus on weight loss. For example, a study of the Unified Treatment Model (UTM)—a transdiagnostic approach to ED treatment—found a more rapid treatment response among the group of individuals with BED, OSFED, and ARFID, compared with the more gradual or static symptoms trajectories seen in the AN and BN group (Espel‐Huynh et al. 2020). Research by Rienecke et al. (2024) examining individuals across the BE spectrum, including BN and OSFED/unspecified feeding and EDs, found that residential treatment incorporating approaches such as cognitive behavioral therapy, acceptance and commitment therapy, dialectical behavioral therapy, and exposure and response prevention led to improvements in ED symptoms and a range of comorbid symptoms, including depression and anxiety, from admission to discharge and at 12‐month follow‐up. Another study by Rienecke et al. (2022) suggested that residential treatment may be particularly beneficial for individuals with BED and a history of trauma, as higher adverse childhood experience scores were associated with greater reductions in BE symptoms during treatment. Additionally, decreases in delay discounting and impulsivity associated with BE behaviors have been reported in residential care (Donahue et al. 2025). However, the study reporting these findings (Donahue et al. 2025) included diagnostically mixed groups and documented BED‐specific outcomes in only a small subset of participants. Overall, while these findings indicate encouraging effects of residential ED treatment for BED, the frequent diagnostic aggregation, small sample sizes, and limited focus on BED‐specific populations constrain understanding of the unique clinical characteristics and treatment outcomes for individuals with BED in residential care.

### 1.2 Transdiagnostic Treatment Approach

BE is a defining feature of BED (APA 2022). Pronounced eating, shape, and weight concerns (Gianini et al. 2013; APA 2022), as well as depressive and anxiety symptoms (Udo and Grilo 2019), often co‐occur with BED. Given the complexity and multidimensionality of BED symptomatology, transdiagnostic treatment approaches that do not focus on weight loss, such as the UTM, may be particularly well‐suited for this population. The UTM is a transdiagnostic protocol implemented at the two residential treatment sites located in the United States examined in the present study. Prior research indicates that the UTM promotes clinical improvement across ED diagnoses (Thompson‐Brenner et al. 2018). Individuals receiving UTM‐based treatment have demonstrated reductions in ED symptom severity, depression, experiential avoidance, anxiety sensitivity, and trauma‐related symptoms, as well as increases in mindfulness (Thompson‐Brenner et al. 2018; Mitchell et al. 2021). However, no published study to date has examined the UTM's effectiveness specifically for individuals with BED.

### 1.3 Current Study

Given the high prevalence of BED and the limited research examining outcomes for this population within residential ED treatment settings, further investigation is needed to better understand the clinical characteristics and treatment responses among individuals with BED in such programs. Considering the frequent comorbidity of BED with other mental health conditions, it is particularly valuable to evaluate how a transdiagnostic model of care may influence both general psychopathology and ED‐specific symptoms.

The purpose of the present study was to examine the clinical outcomes of individuals diagnosed with BED who received treatment within a residential program utilizing the UTM. Specifically, the study investigated changes in ED symptom severity, BE frequency, anxiety symptoms, and depressive symptoms from admission to discharge and at 6‐month follow‐up (6MFU). Based on previous research that individuals with BED may respond rapidly to residential treatment (Espel‐Huynh et al. 2020), and that the UTM is effective at reducing ED, anxiety, and depressive symptoms (Thompson‐Brenner et al. 2019; Mitchell et al. 2021, Emelianchik‐Key et al. 2025), we predicted significant reductions in these symptoms from admission to discharge. It was anticipated that being in the controlled setting of a residential treatment setting would additionally reduce BE frequency between these time points. We also anticipated a rebound of symptoms at the 6MFU, which is typical in ED treatment outcomes (Thompson‐Brenner et al. 2019; Mitchell et al. 2021; Scharff et al. 2021), but we predicted that symptom improvements would still be significantly better than at time of admission.

## 2 Method

### 2.1 Participants

During the treatment center's intake process, patients are provided with the opportunity to consent to having their de‐identified data be used for research. This study was a secondary data analysis of this existing research database maintained by the treatment center. Participants considered for this study from that database were 206 patients who were admitted to either one of The Renfrew Center's residential treatment programs between January 2017 and December 2024 and diagnosed with BED. We then excluded 10 patients due to a length of stay of fewer than 7 consecutive days. An additional 28 participants were excluded due to not completing clinical measures at two or more assessment timepoints (admission, discharge, 6‐month follow‐up). The final analytic sample consisted of 168 participants (see Figure 1 for CONSORT flow diagram).

**FIGURE 1:**
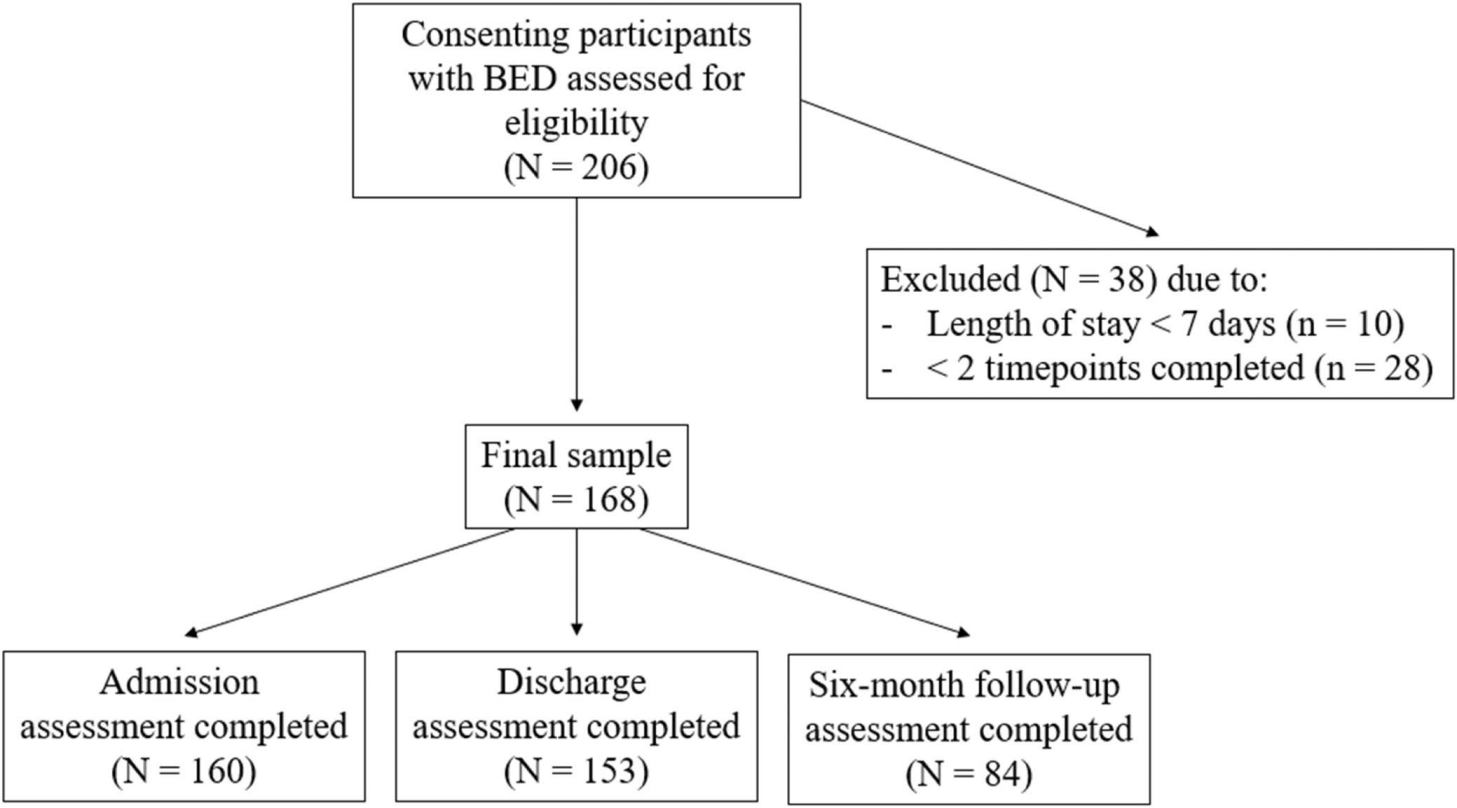
CONSORT flow diagram of participants.

Upon admission, the majority of participants identified as female (99.1% sex assigned at birth and 94.6% gender identity) and White (80.0%), and the average age was 32. The average length of residential stay was 31 days. The most common co‐occurring diagnoses were depressive disorders (*n* = 138, 82.1%), followed by anxiety disorders (*n* = 76, 45.2%), and PTSD (*n* = 60, 35.7%). Detailed participant characteristics at admission can be found in Table 1.

**TABLE 1:** Participant characteristics.

	*n*	%
Sex assigned at birth (*n* = 115)^a^		
Female	114	99.1
Male	1	0.9
Sexual orientation (*n* = 111)		
Heterosexual	78	70.3
Bisexual	14	12.6
Homosexual/gay or lesbian	9	8.1
Questioning	2	1.8
Pansexual	2	1.8
Asexual	1	0.9
Declined to respond	5	4.5
Gender identity (*n* = 93)		
Female	88	94.6
Transgender‐ FTM	2	2.2
Non‐binary	2	2.2
Gender fluid	1	1.1
Race/ethnicity (*n* = 160)		
Caucasian	128	80.0
African American	9	5.6
Hispanic	9	5.6
Multiracial	7	4.4
Native American	1	0.6
Other	6	3.8
Marital status (*n* = 160)		
Single	111	69.4
Married	32	20.0
Divorced	9	5.6
Committed relationship	4	2.5
Separated	3	1.9
Widowed	1	0.6
Education (*n* = 160)		
Less than high school	33	20.6
Completed high school	18	11.3
Some college	32	20.0
Associate's degree (2 year)	10	6.3
Technical/training school	4	2.5
Bachelor's degree (4 year)	45	28.1
Graduate school	18	11.3
Employment (*n* = 160)		
Unemployed	46	28.7
Employed part‐time	18	11.3
Employed full‐time	56	35.0
Student	26	16.3
Working, but not for money (i.e., homemaker, volunteer)	7	4.4
Retired	7	4.4
Residential site location (*n* = 168)		
United States Northeast	112	66.7
United States Southeast	56	33.3

	*M*	SD	Min.	Max.
Age (*n* = 168)	32.14	15.17	14.00	65.00
BMI				
Admission (*n* = 163)	44.44	13.27	20.30	84.80
Discharge (*n* = 141)	43.28	12.96	19.80	82.20
6MFU (*n* = 84)	45.24	12.84	21.14	83.39
Number of BE episodes^b^				
Admission (*n* = 160)	19.63	15.69	1.00	85.00
Discharge (*n* = 153)	2.38	5.10	0.00	28.00
6MFU (*n* = 84)	8.13	9.29	0.00	50.00
Length of residential stay (days; *n* = 168)	31.02	9.74	7.00	71.00

^a^ Self‐report data for gender identity and sex assigned at birth was captured from 2019 to 2024.

^b^ Number of BE episodes refers to the last 28 days prior to response.

### 2.2 Procedure

Trained providers conducted semi‐structured assessments to determine current primary and comorbid diagnoses at time of admission. Patients admitted to Renfrew treatment programs complete standardized clinical measures as part of routine clinical practice. Measures were administered through electronic surveys on iPads at scheduled intervals throughout treatment (i.e., at admission, weekly during care, and at discharge). Participants were also invited via email to complete the measures 6 months after discharging from residential care. All research activities were approved by the internal review board at The Renfrew Center and did not qualify as human subjects research by the University of Michigan Institutional Review Board.

### 2.3 Treatment

Residential treatment at The Renfrew Center includes supervised meals and snacks, structured daily activities, group therapy sessions, and individual sessions with psychotherapists, dieticians, and psychiatrists. Treatment is centered around the UTM, a transdiagnostic standardized model of care (Thompson‐Brenner et al. 2018), which was amended from the Unified Protocol for addressing comorbid emotional disorders to specifically treat EDs and other co‐occurring emotional disorders. The UTM incorporates principles from several modalities including cognitive behavioral therapy, acceptance and commitment therapy, exposure and response prevention, motivational interviewing, and mindfulness. The UTM approaches treatment from a trauma‐informed, relational, and weight‐neutral lens and is a modular treatment that focuses on increasing emotional awareness, reducing emotional avoidance, and improving emotion regulation. The UTM organizes the modules in three phases: (1) motivation enhancement, psychoeducation and treatment rationale, and emotion awareness, tracking, and present‐focused awareness; (2) cognitive appraisals and reappraisals, emotion avoidance and emotion‐driven behaviors, and awareness and tolerance of physical sensations; and (3) situation‐based emotion exposures for food, body image, and co‐occurring emotional distress, and relapse prevention.

### 2.4 Measures

#### 2.4.1 ED Symptoms

The Eating Disorder Examination Questionnaire (EDE‐Q; Fairburn and Beglin 1994, 2008) global score was used to examine overall ED severity. The EDE‐Q is a 28‐item self‐report measure using a 7‐point Likert scale (0–6) and assesses ED symptoms experienced over the 28 days. Global ED severity scores range from 0 to 6, with higher scores indicating greater severity. The EDE‐Q is a widely accepted measure for assessing ED symptoms across ED subtypes, including BED, and shows good psychometric properties of reliability and discriminant validity of ED psychopathology, including high internal consistency and test–retest reliability (Reas et al. 2006; Aardoom et al. 2012; Berg et al. 2012). Within this sample, the measure demonstrated good internal consistency with the global score for admission, discharge, and 6MFU timepoints (*α* = 0.92, *α* = 0.93, and *α* = 0.95, respectively).

#### 2.4.2 Binge‐Eating Episodes

The number of BE episodes was calculated using item 14 from the EDE‐Q that assesses the number of times the person perceived having a sense of loss of control during episodes of overeating over the past 28 days. As loss of control is a primary criterion for BED diagnosis (APA 2023), including this with overeating episodes may better capture true BE experiences for participants. The EDE‐Q includes behavioral items that assess objective BE episodes, showing robust test–retest reliability and construct validity for objective BE episodes (Berg et al. 2012).

#### 2.4.3 Anxiety Symptoms

Anxiety symptoms were assessed using the Overall Anxiety Severity and Impairment Scale (OASIS; Norman et al. 2006). The OASIS is a 5‐item self‐report measure of frequency, intensity, and functional impairment over the past week scored on a 5‐point Likert scale with a total possible score ranging from 0 to 20 with higher scores indicating worse anxiety symptoms. The scale shows strong psychometric properties with good internal consistency, test–retest reliability, and convergent/discriminant validity (Campbell‐Sills et al. 2010; Norman et al. 2006). Clinically significant anxiety cutoffs have been established with a score of 8 or greater (Campbell‐Sills et al. 2010). In our sample, the measure demonstrated good internal consistency across timepoints (admission *α* = 0.85, discharge *α* = 0.88, 6MFU *α* = 0.86).

#### 2.4.4 Depressive Symptoms

Two different measures were used throughout the data collection period to assess depressive symptoms. The Center for Epidemiological Studies Depression Scale (CES‐D; Radloff 1977) was employed from January of 2017 until December of 2022, and the Patient Health Questionnaire (PHQ‐9; Kroenke et al. 2001) was adopted as the new measure of depression starting January of 2023. Patients completed the same depressive measure at each time point. The CES‐D is a 20‐item self‐report measure with response options on a 4‐point Likert scale of agreement with each given statement of depressive symptoms of rarely or none of the time (scored 0) to most or all of the time (scored 3), with a total score ranging from 0 to 60 and a higher score indicating more symptomology. The PHQ‐9 is a 9‐item brief scale using a Likert 4‐point scale of 0 (not at all) to 3 (nearly every day), measuring depressive symptoms over the past 2 weeks with a possible total score ranging from 0 to 27.

The CES‐D and PHQ‐9 show similarities in measuring depression symptoms with similar and robust internal consistency reliability, test–retest reliability, and clinical correlates (Kroenke et al. 2001; Milette et al. 2010). Within this study, internal consistency was good to excellent for the CES‐D and PHQ‐9 at admission, discharge, and 6MFU (*α* = 0.87, *α* = 0.87, and *α* = 0.90; *α* = 0.90, *α* = 0.92, and *α* = 0.91, respectively). The CES‐D and PHQ‐9 show similar performance of indicating depressive symptoms, showing good convergent validity (Cook et al. 2017; Kim et al. 2015; Milette et al. 2010).

A composite depression index was created for analyses, averaging and standardizing CES‐D and PHQ‐9 scores with z‐scores. Z‐score transformed depression scores ranged from −2.19 to 2.41. Higher scores indicated greater depressive symptoms.

#### 2.4.5 Body Mass Index (BMI)

BMI was calculated as (weight [lb]/height [in]^2^ × 703). Height and weight are collected by nursing staff on the day of admission into programming and typically within 2 days of discharging from programming.

### 2.5 Analysis

Analyses were conducted using SPSS version 29 (IBM Corp 2025). Data completeness varied by timepoint, with increasing missingness observed at admission (4.8%), discharge (8.5%–9.5%), and 6MFU (50%). While this degree of attrition at follow‐up is typical in naturalistic treatment contexts, independent‐samples *t*‐tests were conducted to evaluate group differences between participants who completed two versus three assessments on baseline demographic (age, race, gender, BMI, and site) and clinical (BE episodes and ED, anxiety, and depressive symptoms) variables. Results indicated that participants who did not complete three assessments were more likely to identify as non‐White (*t* = 2.67, *p* = 0.01) and had a lower BMI at admission (*t* = −1.99, *p* = 0.04).

A non‐significant Little's Missing Completely at Random (MCAR) test indicated that missingness was at random (MAR). To further evaluate the MAR assumption, we examined whether baseline demographic and clinical variables predicted missing status in terms of eligibility. No baseline variables significantly predicted eligibility (see Table 2). Next, we tested whether baseline variables predicted the missing status of data of those who completed two versus three assessments. Higher BMI at admission was associated with a greater likelihood of completing three versus two assessments (see Table 2). Taken together, these findings support the assumption that data were mostly MAR.

**TABLE 2:** Logistic regression analyses examining baseline predictors of eligibility and number of assessments completed.

Predictor	Eligibility						Assessments completed					
	*B*	SE	Wald *χ* ^2^	OR	95% CI	*p*	*B*	SE	Wald *χ* ^2^	OR	95% CI	*p*
Age	0.03	0.02	1.28	1.03	[0.98, 1.08]	0.26	−0.04	0.02	2.97	0.96	[0.92, 1.01]	0.09
EDE‐Q global score	0.12	0.25	0.24	1.13	[0.69, 1.86]	0.62	−0.19	0.29	0.41	0.83	[0.47, 1.47]	0.52
OASIS total score	0.00	0.08	0.00	1.00	[0.85, 1.17]	0.99	−0.04	0.08	0.33	0.96	[0.82, 1.11]	0.57
Depression *Z* score	−0.31	0.51	0.24	0.80	[0.30, 2.07]	0.63	−0.26	0.49	0.23	0.79	[0.30, 2.07]	0.63
BMI at admission	0.02	0.02	0.95	1.02	[0.98, 1.07]	0.33	0.05	0.03	4.34	1.06	[1.00, 1.11]	**0.04** *
Binge‐eating episodes	−0.01	0.03	0.03	0.99	[0.94, 1.05]	0.85	0.06	0.04	0.08	1.01	[0.94, 1.09]	0.78

*Note:* Outcome coded as 0 = eligible or two assessments completed and 1 = not eligible or three assessments completed. Bolded *p* value indicates significance.

Abbreviations: CI = confidence interval; EDE‐Q = Eating Disorder Examination Questionnaire; OASIS = Overall Anxiety Severity and Impairment Scale; OR = odds ratio.

* *p* < 0.05.

Linear mixed‐effects models (LMMs) were conducted separately for each of the four primary outcomes to evaluate changes in clinical outcomes across the three timepoints. LMMs are widely used in clinical longitudinal studies to evaluate mean differences, providing a robust and flexible analytic framework for repeated‐measures data and accommodating correlated observations and incomplete data under the MAR assumption (Gueorguieva and Krystal 2004; Hedeker and Gibbons 2006). This approach allows for simultaneous estimation of fixed effects (e.g., time) and random effects (e.g., individual variability), providing unbiased estimates in the presence of missing data and non‐sphericity (Arnau, Bono, and Vallejo 2012; Arnau, Bono, Blanca, and Bendayan 2012; Schielzeth et al. 2020).

Preliminary assumption checks confirmed the suitability of LMM estimation. For the EDE‐Q Shapiro–Wilk tests indicated deviations from normality at admission and discharge (*p* < 0.05); however, model residuals of the composite timepoints met normality (*p* > 0.05), with skewness and kurtosis values (−0.81 to 0.0.54; −0.53 to 0.24) falling within acceptable ranges for psychological data (Schielzeth et al. 2020). These results supported untransformed analyses for the EDE‐Q consistent with established guidelines for repeated‐measures models (Gueorguieva and Krystal 2004). Frequency of BE episodes exhibited significant right‐skewness and kurtosis of the composite timepoints (skewness = 2.33, SE = 0.12; kurtosis = 8.55, SE = 0.24; both *p*s < 0.001), so logarithmic transformation via ln(*x* + 1) was applied to normalize the distribution and stabilize variance prior to running the LMM, recommended for zero‐inflated count data in longitudinal designs (Feng et al. 2014). Estimated marginal means (EMM) and pairwise differences from this model were then back‐transformed to the original scale for interpretation. Anxiety symptoms and depressive symptoms met normality assumptions and did not show significant outliers affecting the data. All model residuals showed an even spread of data with no visible heteroscedasticity.

LMMs were estimated using restricted maximum likelihood (REML) estimation to account for unbalanced longitudinal data with missing observations (West et al. 2014; Verbeke and Molenberghs 2000). For each outcome, time (three levels: admission, discharge, and 6MFU) was entered as a fixed categorical predictor (effect), and a random intercept for participant ID was included to account for within‐subject dependence. Age, BMI at admission, and site were included as covariates in preliminary models as these variables have been shown to influence ED residential treatment outcomes (e.g., Scharff et al. 2021). However, BMI and age were not significant predictors of any of the dependent variables in this study. The LMM models were then run without those two covariates, as is recommended to reduce overfitting, and improve interpretability for parsimony of information (Seltman 2012). Multiple covariance structures for the repeated‐measures component were compared. A compound symmetry (CS) covariance structure was retained for the final models, providing stable estimation of fixed effects. Mild non‐normality and skew of the EDE‐Q and unbalanced data due to missingness between time points across measures were further accounted for via the Kenward–Roger correction, enhancing precision of parameter estimates and *F*‐tests by adjusting degrees of freedom for conservative estimates (Arnau, Bendayan, et al. 2013; Arnau, Bono, and Vallejo 2013; Luke 2017). EMM with Bonferroni‐adjusted pairwise comparisons were computed to control for multiple pairwise comparisons and interpret changes in scores over time. Effect sizes were quantified as partial eta‐squared (*η*_p_^2^) derived from the Type III F‐tests for timepoint, interpreted per Cohen's (1988) conventions (small: ≥ 0.01; medium: ≥ 0.06; large: ≥ 0.14). Within‐group effect sizes (Cohen's *dz*) were also calculated with 95% confidence intervals, corrected for correlated observations. Effect sizes were interpreted using the following conventional cutoffs: small (*dz* ≥ 0.2), medium (*dz* ≥ 0.5), and large (*dz* ≥ 0.8).

## 3 Results

Longitudinal changes in ED symptoms, anxiety symptoms, depressive symptoms, and BE frequency indicated significant improvement from admission to discharge, with partial rebound at 6MFU; however, still lower than at admission. Findings support a large overall effect of time (*η*_p_^2^ > 0.40, *p* < 0.001 for all models, see Table 3).

**TABLE 3:** Estimated marginal means and pairwise comparisons across timepoints.

Measure	Timepoint	EMM	SE	95% CI	Contract	(*M*_diff_)	Cohen's *dz*	95% CI
EDE‐Q global	Admission (T1)	3.52	0.09	[3.34, 3.71]	T1‐T2	1.41***	1.36	[1.14, 1.59]
	Discharge (T2)	2.11	0.08	[1.94, 2.27]	T1‐T3	0.64***	0.48	[0.31, 0.65]
	6‐Month Follow‐Up (T3)	2.88	0.13	[2.63, 3.14]	T2‐T3	−0.76***	−0.59	[−0.76, −0.41]
Oasis total	Admission (T1)	12.25	0.33	[11.61, 12.91]	T1‐T2	4.21***	1.04	[0.83, 1.24]
	Discharge (T2)	8.05	0.32	[7.41, 8.70]	T1‐T3	2.80***	0.69	[0.51, 0.87]
	6‐Month Follow‐Up (T3)	9.46	0.43	[8.60, 10.32]	T2‐T3	−1.37***	−0.37	[−0.54, −0.20]
Standardized depression score	Admission (T1)	0.60	0.07	[0.46, 0.73]	T1‐T2	1.06***	1.16	[0.94, 1.37]
	Discharge (T2)	−0.46	0.07	[−0.60, −0.32]	T1‐T3	0.86***	0.78	[0.59, 0.97]
	Follow‐up (T3)	−0.27	0.09	[−0.45, −0.08]	T2‐T3	−0.19	−0.13	[−0.30, 0.04]

	Timepoint	Back‐transformed EMM	95% CI lower	95% CI upper	Contract	% Change	Cohen's *dz*	95% CI
Number of BE episodes	Admission (T1)	13.57	11.53	15.96	T1‐T2	−86%***	0.37	[0.20, 0.53]
	Discharge (T2)	0.98	0.7	1.31	T1‐T3	−62%***	0.16	[0.01, 0.32]
	6‐Month Follow‐up (T3)	4.48	3.46	5.73	T2‐T3	+177%***	−0.69	[−0.87, −0.51]

*Note:* EMM = estimated marginal mean; SE = standard error; CI = confidence interval; *M*_diff_ = mean difference; % Change = back‐transformed percentage difference (negative = reduction; positive = increase). Number of BE Episodes were ln(*x* + 1)‐transformed for analysis; values are approximate counts (medians).

*** *p* < 0.001.

### 3.1 ED Symptoms

EMMs and pairwise comparisons indicated a significant and large decline from admission (*M* = 3.52) to discharge (*M* = 2.11, *p* < 0.001; Cohen's *dz* = 1.36) and a significant and small decline from admission to 6MFU (*M* = 2.88, *p* < 0.001; Cohen's *dz* = 0.48). However, scores rose significantly with a medium effect from discharge to follow‐up (mean difference = −0.76, *p* < 0.001; Cohen's *dz* = −0.59), suggesting partial symptom resurgence.

### 3.2 Anxiety Symptoms

Anxiety symptoms decreased largely from admission (*M* = 12.25) to discharge (*M* = 8.05, *p* < 0.001; Cohen's *dz* = 0.1.04). Scores at 6MFU (*M* = 9.46) were significantly lower than at admission with a medium effect size (*p* < 0.001; Cohen's *dz* = 0.69) but higher than at discharge (mean difference = −1.37, *p* = 0.004; Cohen's *dz* = −0.37), indicating a small rebound.

### 3.3 Depressive Symptoms

The model for the standardized depression score (z‐transformed) yielded a significant and large timepoint effect. Scores significantly and largely decreased from admission (*M* = 0.60) to discharge (*M* = −0.46; *p* < 0.001; Cohen's *dz* = 1.06) and from admission to 6MFU (*M* = −0.27; *p* < 0.001; Cohen's *dz* = 0.78). The difference between discharge and follow‐up was nonsignificant (*p* = 0.128; Cohen's *dz* = −0.13), indicating stability of lower depressive scores.

### 3.4 Binge‐Eating Episodes

For the number of BE episodes over the past month, back‐transformed EMMs decreased from 13.57 episodes at admission to 0.98 at discharge which was a small effect size (86% reduction, *p* < 0.001; Cohen's *dz* = 0.37) and 4.48 at 6MFU (62% reduction from admission, *p* < 0.001; Cohen's *dz* = 0.16). Episodes increased significantly from discharge to follow‐up with a medium effect size (177% increase, *p* < 0.001; Cohen's *dz* = −0.69). These back‐transformed values approximate adjusted counts.

## 4 Discussion

The current study fills a long‐standing gap in the literature by examining the changes in ED and comorbid symptoms for those diagnosed with BED at time of admission to discharge, and 6 months following residential ED treatment. Our findings demonstrated that individuals with BED experienced clinically meaningful and statistically significant improvement across ED symptoms, BE frequency, anxiety, and depressive symptoms from admission to discharge. Further, these gains were largely sustained at 6MFU, although with some partial symptom resurgence for ED psychopathology, anxiety, and BE frequency. While the study lacked a control group and thus the change in symptoms cannot be solely attributed to the treatment, results align with outcomes reported for other ED diagnoses treated in residential care, and build upon the very limited existing evidence on ED treatment residential outcomes specific for individuals with BED (e.g., Thompson‐Brenner et al. 2019; Espel‐Huynh et al. 2020; Peckmezian and Paxton 2020).

The mean frequency of BE episodes at admission (19.63 episodes) was higher than that reported in outpatient treatment studies (e.g., Anderson et al. 2020 reported a mean of 15.8 episodes over the past 4 weeks), reflecting greater clinical severity and the need for a higher level of care among these patients. BE frequency also demonstrated the most significant change across time points. While the dramatic decrease in frequency at time of discharge is likely reflective of the structured nature of residential treatment and reduced opportunity to engage in ED behaviors, the 62% reduction in BE from baseline to 6MFU has significant clinical implications. At time of admission, the mean BE frequency of 19.6 episodes in a 4‐week period would be categorized as moderate severity, while the mean frequency of eight BE episodes over a 4‐week period at 6MFU would be categorized as mild severity in the DSM‐5‐TR (APA 2022). Previous work has found meaningful differences between these severity categories in level of psychological distress, social maladjustment and illness‐specific functional impairment (Dakanalis et al. 2017; Grilo et al. 2015). Thus, a categorical reduction in severity likely increases the quality of life of these patients and may increase the likelihood of recovering from their ED (e.g., Fischer et al. 2014).

Significant decreases were also observed in global ED psychopathology and anxiety, with some rebound at time of 6MFU. This pattern is consistent with previous ED residential research (e.g., Clague et al. 2024; Day et al. 2025; Thompson‐Brenner et al. 2019). Uniquely, depressive symptoms exhibited a steep improvement during treatment and remained stable rather than rebounding at follow‐up. This is important given the high comorbidity with depressive symptoms and that previous research has found that depression can mediate the association between BED and health‐related quality of life, even when controlling for weight status (Singleton et al. 2019). Although the present study did not include a control condition, we sought to contextualize our findings by comparing our observed effect sizes with those reported for outpatient treatment for BED. Effect sizes from admission to discharge in the current study appeared larger for ED (1.41 versus 0.79), anxiety (4.21 versus 0.24), and depressive symptoms (1.06 versus 0.37) than those reported in outpatient therapy meta‐analyses (Cuijpers et al. 2025; Zhong et al. 2024). While these comparisons should be interpreted cautiously and future work should include a control group to better understand treatment effects, these findings underscore that individuals with BED may respond robustly to residential ED treatment that is transdiagnostic and emotion‐focused, and symptom improvements remain significant and meaningful at time of follow‐up.

Finally, the present analyses did not identify age or BMI as covariates that predicted differential treatment response. Future research should investigate these factors, particularly BMI, to better understand if weight is a factor in treatment response, since BMI is a considered factor in treatment access by health insurance and within utilization‐management policies. Future research should also examine whether other clinical or psychosocial factors, such as length of stay and insurance/payment method, moderate treatment response.

### 4.1 Clinical Implications

The UTM may be uniquely suited for treating BED due to its transdiagnostic focus on emotion awareness, cognitive reappraisal, exposure to body‐ and food‐related distress, and emotion‐driven behavior reduction. These mechanisms map onto well‐established antecedents of BE behaviors, including heightened negative affect, maladaptive emotion regulation, and internalized weight stigma (e.g., Svaldi et al. 2019; Hayward et al. 2018). Accordingly, the high dose and immersive nature of residential treatment may accelerate exposure‐based learning and strengthen behavioral alternatives to BE, explaining both large reductions at discharge and maintenance of most gains at follow‐up. The observed rebounds at follow‐up highlight a potential need for extended step‐down support targeting ED psychopathology, BE, and anxiety. Future research should investigate stepped‐care models, including partial hospitalization programs, intensive outpatient programs, and structured booster sessions, as tools to help prevent relapse.

### 4.2 Strengths and Limitations

A significant strength of this study is that it is the largest known multi‐site examination of residential outcomes for individuals with BED to date. Further, we were able to examine change in multiple clinically relevant symptoms across three time points. The study had several limitations, including a modest follow‐up survey response rate, which is typical of naturalistic longitudinal treatment research. Additionally, information on the types of treatments or care settings patients engaged in following discharge from residential treatment was unavailable, which limits interpretation of symptom change at follow‐up. We also relied on self‐report measures rather than clinician‐rated outcomes. The sample was homogenous in gender identity/sex assigned at birth, race/ethnicity, and sexual orientation, typical for ED residential care settings; however, it inadequately reflects the general population of individuals with BED (e.g., Cheng et al. 2019). Finally, the naturalistic design precludes causal inference regarding specific treatment components.

## 5 Conclusion

Despite the prevalence of BED, individuals with this diagnosis seldom access residential ED treatment and outcomes for this level of care have been understudied. This study provides compelling evidence that a UTM‐based residential ED treatment produces substantial improvement in BE behaviors, ED psychopathology, anxiety, and depressive symptoms among individuals with BED. Although some symptom rebound occurred after discharge, outcomes suggest that residential care represents a powerful, although often under‐utilized, treatment avenue for BED, particularly when care is grounded in an emotion‐focused, weight‐neutral, transdiagnostic treatment model. Continued investment in improving access to residential ED care and research into stepped‐down continuity of care may strengthen long‐term outcomes and reduce barriers to care for this highly prevalent ED.

## Author Contributions

**Kiki M. Kline:** conceptualization, formal analysis, investigation, methodology, writing – review and editing, writing – original draft, project administration. **Briana Delibashi:** data curation, writing – review and editing. **Gayle Brooks:** supervision, resources, writing – review and editing. **Shelby N. Ortiz:** writing – original draft, writing – review and editing, supervision, conceptualization, formal analysis.

## Lived Experience Involvement Statement

No specific efforts were undertaken to involve persons with lived experience in the study design or execution, or in the preparation of this manuscript.

## Funding

The authors have nothing to report.

## Disclosure

The authors are employees of The Renfrew Center.

## Conflicts of Interest

The authors declare no conflicts of interest.

## Data Availability

The data that support the findings of this study are available from the corresponding author upon reasonable request.
